# Pruritus in patients with chronic liver disease and serum autotaxin levels in patients with primary biliary cholangitis

**DOI:** 10.1186/s12876-019-1092-z

**Published:** 2019-10-24

**Authors:** Hatsue Fujino, Mio Tanaka, Michio Imamura, Kei Morio, Atsushi Ono, Takashi Nakahara, Eisuke Murakami, Tomokazu Kawaoka, Shoichi Takahashi, Daiki Miki, Masataka Tsuge, Akira Hiramatsu, Hiroshi Aikata, C. Nelson Hayes, Kazuaki Chayama

**Affiliations:** 1 Department of Gastroenterology and Metabolism, Graduate School of Biomedical and Health Science, Hiroshima University, 1-2-3 Kasumi, Minami-ku, Hiroshima, 734-8551 Japan; 2 Research Center for Hepatology and Gastroenterology, Hiroshima University, Hiroshima, Japan; 3 Natural Science Center for Basic Research and Development, Hiroshima University, Hiroshima, Japan

**Keywords:** Pruritus, Numerical rating scale, Primary biliary cholangitis, Autotaxin

## Abstract

**Background:**

Pruritus is a common symptom seen in patients with chronic liver disease. However, frequency and severity of pruritus in patients with chronic liver disease is unclear. We investigated frequency, severity and predictive factors of pruritus in these patients from a large cohort.

**Methods:**

A total of 2477 patients with chronic liver disease without allergies or skin diseases were investigated for itch frequency and severity. Itch severity was self-assessed using pruritus scores using the numerical rating scale (NRS). Multivariate regression analysis was performed to identify factors associated with pruritus. Serum autotaxin levels were measured in patients with primary biliary cholangitis (PBC), and the relationship to liver fibrosis and pruritus was analyzed.

**Results:**

The frequency of pruritus in patients with chronic liver disease was significantly higher than in subjects without liver disease (29.8 and 16.2%, respectively, *P* < 0.001). NRS was high in patients with chronic liver disease, especially in those with PBC, as is generally expected. Multivariate analysis identified lower albumin, higher eosinophil count, and etiology of PBC as independent factors associated with severe pruritus (≥5 points of NRS). In patients with PBC, serum autotaxin levels were significantly correlated with liver fibrosis markers such as platelet count and liver stiffness, and hepatobiliary enzymes such as total bilirubin, aspartate aminotransferase and alkaline phosphatase. However, no significant correlations between serum autotaxin levels and frequency and severity of pruritus were observed in patients with PBC.

**Conclusion:**

The frequency of pruritus was high in patients with chronic liver disease. Reduction of liver function is associated with severe pruritus based on the large number of patients with chronic liver disease. Serum autotaxin is useful for assessing liver fibrosis and severity of cholangitis; however, it is not a predictive marker for severe pruritus in patients with PBC.

## Background

Pruritus is defined as an unpleasant sensation that triggers the need to scratch [1]. It develops in association with chronic liver diseases, including hepatitis, liver cirrhosis, primary biliary cholangitis (PBC) and obstructive jaundice. Pruritus associated with chronic liver disease tends to be generalized and is not relieved by scratching [2], with marked negative impact on the quality of life of the patients [3]. The intensity of pruritus does not correlate with biochemical indices of liver disease; thus, several factors might contribute directly or indirectly to pruritus [4]. However, factors associated with severe pruritus in patients with chronic liver disease are not well known. Although several therapeutic options such as cholestyramine, antihistamines, hypnotics, ursodeoxycholic acid, anti-allergic agents, and nalfurafine hydrochloride are available for pruritus [5–8], the efficacy of each of these drugs is limited. Therefore, establishment of useful prediction markers for pruritus in chronic liver disease is required.

Autotaxin is a secreted enzyme originally discovered in conditioned medium from A2058 human melanoma cell cultures [9] and has important functions in converting lysophosphatidylcholine to lysophosphatidic acid [10]. Serum autotaxin levels increase with progression of liver fibrosis caused by various etiologies such as chronic hepatitis C, chronic hepatitis B, non-alcoholic steatohepatitis (NASH), and PBC [11–15], and serum autotaxin level is also associated with pruritus in patients with cholestasis [16].

In the present study, we undertook an interview-based survey and investigated the frequency and severity of pruritus and factors associated with severe pruritus based on a large number of patients with chronic liver disease. We also analyzed the relationship between serum autotaxin levels and liver fibrosis and pruritus in patients with PBC.

## Methods

### Study design and participants

A total of 2477 patients with chronic liver disease who visited Hiroshima University Hospital from 2016 to 2017 were retrospectively investigated for itch frequency and severity. Characteristics of the 2477 patients with chronic liver disease at the time of evaluation of pruritus are shown in Table 1. The study included 1239 males and 1238 females, aged 20 to 96 years (median, 67 years). Seven hundred eighteen patients (29.0%) were positive for hepatitis B surface antigen (HBsAg) (Chemiluminescent Enzyme Immunoassay; Abbott Laboratories, Tokyo, Japan). 1114 (45%) were positive for hepatitis C virus (HCV) antibody (third generation enzyme immunoassay; Chiron, Emeryville, CA, USA). Seven hundred eighty-five out of 1114 HCV antibody-positive patients had received anti-HCV therapies before this survey, out of which 775 had achieved sustained viral response. One hundred two patients (4.1%) had autoimmune hepatitis (AIH), diagnosed according to the criteria established by the International Autoimmune Hepatitis Group (IAIHG). One hundred nineteen patients (4.8%) had PBC, diagnosed according to the criteria established by the Intractable Hepato-Biliary Diseases Study Group of Japan. Two hundred eighty-nine patients (11.7%) had non-alcoholic fatty liver disease (NAFLD), diagnosed based on existence of hepatic steatosis, either by imaging or histology and exclusion of secondary causes of liver steatosis as alcohol, viral hepatitis, autoimmune disorder, and hereditary liver disease. One hundred thirty-five patients (5.5%) had alcohol liver damage, diagnosed based on chronic alcohol use presenting with liver disease and excluding other secondary causes of liver disease. Patients who had complications due to allergic or skin diseases assessed by medical interviews were excluded from this study. Of all patients, 384 patients (15.5%) had been treated with topical cream and/or oral antipruritic agents such as hydrocortisone, antihistamines, kappa-opioid receptor agonists, and moisturizers. Itch frequency was also investigated in 130 control subjects, including healthy subjects (subjects without liver disease) and patients without liver disease. FIB-4 index was calculated as a surrogate marker of liver fibrosis: age [years] × aspartate aminotransferase (AST) [U/L] / platelet counts [10^9^/L] × alanine aminotransferase [U/L]^1/2^ [17]. All participants provided written informed consent to participate in the study according to the process approved by the ethical committee of Hiroshima University and conforming to the ethical guidelines of the Declaration of Helsinki.

**Table 1:** Factors associated with severe pruritus (NRS ≥5 points)

	NRS < 5 (*n* = 2160)	NRS ≥5 (*n* = 317)	Univariate analysis	Multivariate analysis	
			*P* value	OR (95% CI)	*P* value
Sex (male/female)	1074/1086	165/152	0.439		
Age (<65/> 65)	911/1249	141/176	0.438		
Leukocyte count (/mm^3^)	5320 (1600–22,510)	5250 (1900–19,720)	0.857		
Hemoglobin (g/dL)	13.5 (6.5–17.9)	13.3 (5.3–17.5)	0.003		
Platelet count (× 10^4^/μL)	17.8 (2.4–59.5)	17.7 (2.8–51.1)	0.576		
Eosinophil count (/mm^3^)	120 (0–2210)	140 (0–1260)	0.003	1.001 (1.000–1.002)	0.002
Total bilirubin (mg/dL)	0.8 (0.2–16.4)	0.7 (0.2–7.4)	0.256		
Aspartate aminotransferase (IU/L)	24 (7–1162)	25 (5–171)	0.055		
Alanine aminotransferase (IU/L)	19 (1–324)	20 (3–303)	0.387		
γ-glutamyl transpeptidase (IU/L)	23 (7–1362)	27.5 (8–505)	0.003		
Albumin (g/dL)	4.3 (1.2–5.1)	4.2 (2.0–5.1)	< 0.001	0.637 (0.516–0.787)	<0.001
Estimated glomerular filtration rate (mL/min/1.73 m^2^)	71.5 (3.9–192)	70.0 (3.6–181)	0.022		
HbA1c (%)	5.9 (3.5–12.7)	5.8 (3.9–8.7)	0.063		
FIB4 index	2.02 (0.14–51.16)	2.01 (0.34–331.61)	0.431		
Etiology of liver disease					
HBV/others	636/1524	82/235	0.190		
HCV/others	979/1181	135/182	0.360		
AIH/others	91/2069	11/306	0.534		
PBC/others	94/2066	25/292	0.006	2.065 (1.255–3.395)	0.004
NAFLD/others	245/1915	44/273	0.189		
Alcohol/others	115/2045	20/297	0.471		
History of hepatocellular carcinoma (with/without)	499/1661	73/244	0.977		
History of hepatic encephalopathy or varices (with/without)	29/2131	12/305	0.002		

Categorical data are represented as numbers of patients, and continuous data are represented as the median and range

*HBV* hepatitis B virus, *HCV* hepatitis C virus, *AIH* autoimmune hepatitis, *PBC* primary biliary cholangitis, *NAFLD* non-alcoholic fatty liver disease

### Assessment of itch severity

Itch severity was self-assessed using pruritus scores based on a scale ranging from 0 (no itch) to 10 (maximum itch) using the numerical rating scale (NRS) [18]. All 2477 patients with chronic liver disease were evaluated by their pruritus scores.

### Measurement of autotaxin

Serum autotaxin levels were measured in 114 out of 119 PBC patients. Five PBC patients were removed due to lack of sufficient serum samples. Serum autotaxin antigen concentration was determined in frozen serum samples with a specific two-site enzyme immunoassay using AIA-360 system (Tosoh, Tokyo, Japan), as described previously [19].

### Liver stiffness evaluation by FibroScan

Liver stiffness evaluation was performed using FibroScan (Echosens, Paris, France) with a standard probe (M probe). FibroScan measurements were carried out by three independent and experienced observers, blinded with respect to patient data. The objective was to obtain at least ten acceptable measurements. Results were expressed as the median and the IQR (kPa) of all valid measurements. According to the usual definition, liver stiffness was considered reliable when it included ≥10 valid measurements with a success rate ≥ 60% and IQR/M ≤ 0.30. Only reliable results were used for analysis.

### Statistical analysis

The Chi-squared and Mann–Whitney U-tests were applied to detect significant associations. All statistical tests were two-sided, and *P* < 0.05 was considered significant. Correlation analysis was performed using Spearman’s rank-order correlation. Variables that achieved statistical significance (*P* < 0.05) on univariate analysis were entered into multiple logistic regression analysis to identify independent predictors of severe pruritus. All statistical analysis was performed using BellCurve for Excel ver.2.15 (Social Survey Research Information Co., Ltd.).

## Results

### Pruritus in patients with chronic liver disease

**Fig. 1:**
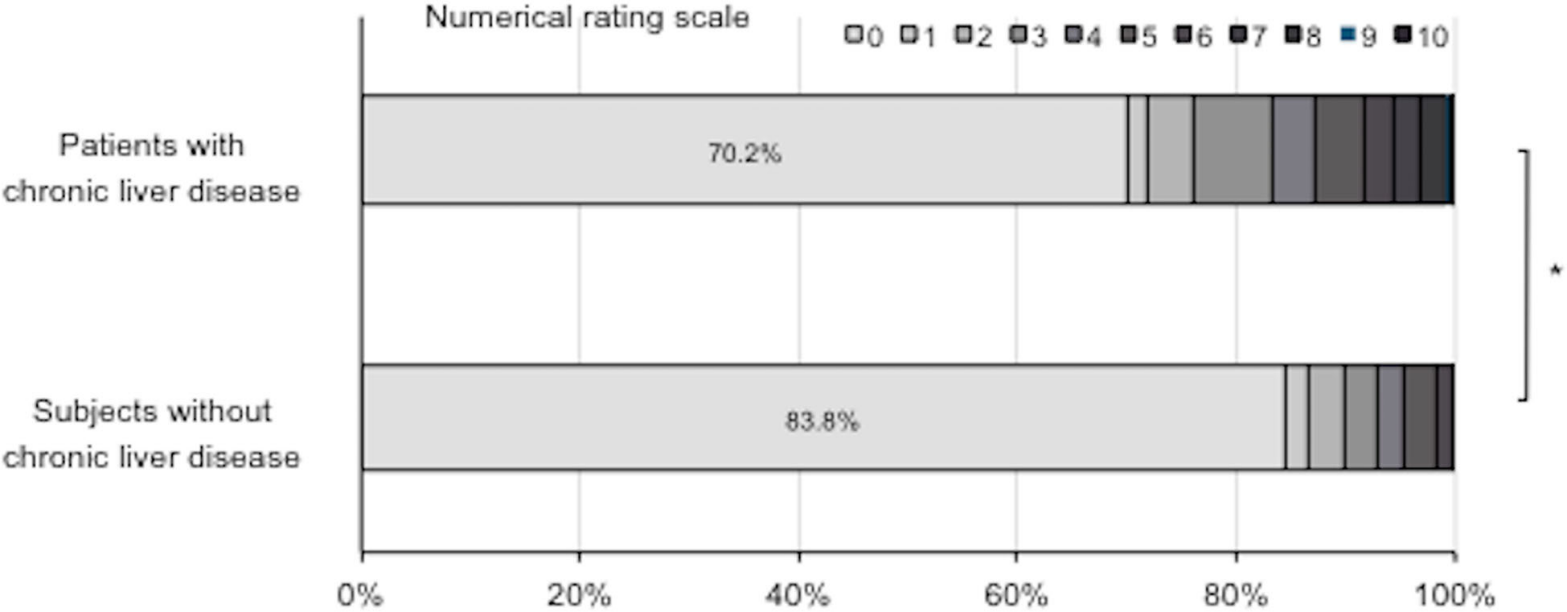
Distribution of numerical rating scale scores in patients with chronic liver disease. Scores in subjects without liver disease were also analyzed. (*, *P* < 0.001)

**Fig. 2:**
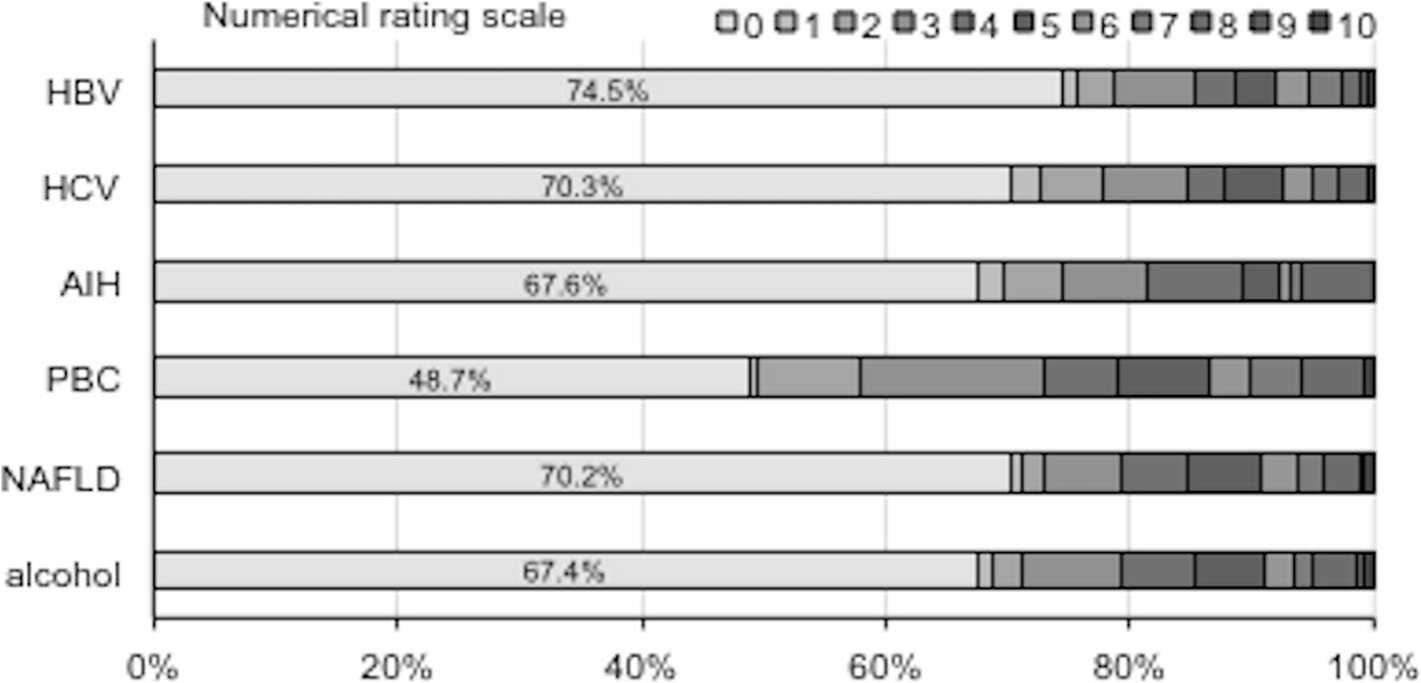
Distribution of NRS pruritus scores according to the etiology of chronic liver disease. NRS scores were high in patients with chronic liver disease, especially among those with PBC

Seven hundred thirty-eight patients (29.8%) were evaluated as having ≥1 point pruritus severity by NRS (Fig. 1). The frequencies of pruritus in patients with chronic liver disease were significantly higher than those in subjects without liver disease (16.2%) (*P* < 0.001). Figure 2 shows the distribution of pruritus scores according to the etiology of chronic liver disease. The average NRS scores for HBV, HCV, AIH, PBC, NAFLD and alcohol were 1.15, 1.23, 1.38, 2.23, 1.43 and 1.51, respectively. NRS was high in patients with chronic liver disease, especially in those with PBC, as is generally expected.

### Factors associated with pruritus

We analyzed factors associated with severe pruritus, which we define as NRS ≥5. Univariate analysis showed that hemoglobin (*P* = 0.003), eosinophil count (*P* = 0.003), γ-glutamyl transpeptidase (*P* = 0.003), albumin (*P* < 0.001), estimated glomerular filtration rate (*P* = 0.022), PBC (*P* = 0.006), and hepatic encephalopathy or esophageal varices (*P* = 0.002) were each significantly associated with pruritus (Table 1). Multivariable analysis identified higher eosinophil count (OR = 1.001; *P* = 0.002), lower albumin levels (OR = 0.637; *P* < 0.001), and PBC (OR = 2.065 for others; *P* = 0.004) as independent factors associated with severe pruritus.

### Serum autotaxin levels and factors associated with pruritus in PBC patients

**Fig. 3:**
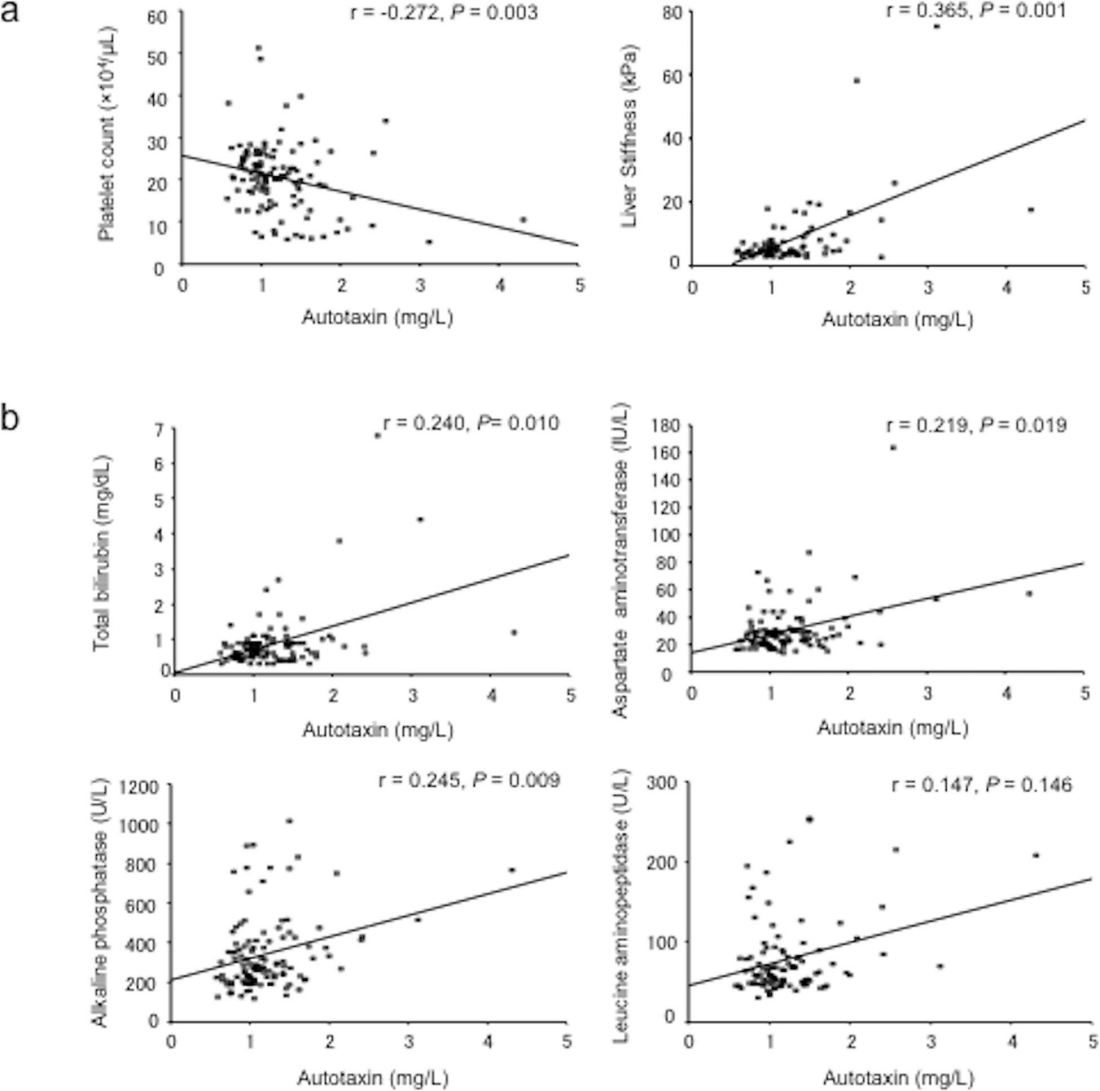
The relationship between autotaxin and liver fibrosis markers and laboratory data in patients with PBC. Significant correlations were observed between serum autotaxin values and (**a**) platelet count and liver stiffness, and (**b**) alkaline phosphatase, total bilirubin, and aspartate aminotransferase

Severe pruritus (NRS ≥5) was reported more frequently in PBC patients. Therefore, we primarily focused on PBC patients in this study. A recent study demonstrated that serum autotaxin was correlated with the degree of liver fibrosis in PBC patients [20], and serum autotaxin levels are known to be high in cholestasis patients with pruritus [16]. Therefore, we analyzed the relationship between serum autotaxin level and liver fibrosis and pruritus in PBC patients. Serum autotaxin levels were measured in 114 out of 119 PBC patients (91 patients were NRS < 5 and 23 patients were NRS ≥ 5). Significant correlations between serum autotaxin values and liver fibrosis markers such as platelet count and liver stiffness were observed (Fig. 3a). Serum autotaxin values were also significantly associated with levels of hepatobiliary enzymes such as total bilirubin, AST, and alkaline phosphatase (Fig. 3b). The relationship between autotaxin levels and pruritus was analyzed; however, autotaxin levels were not correlated with severity of pruritus. In patients with PBC, no factor was significantly correlated with severe pruritus (Table 2).

**Table 2:** Factors associated with severe pruritus (≥5 points of NRS) in PBC patients

	NRS < 5 (*n* = 94)	NRS ≥5 (*n* = 25)	Univariate analysis
			*P* value
Sex (male/female)	14/80	4/21	0.891
Age (< 65/≥65)	49/45	10/15	0.281
Leukocyte count (/mm^3^)	5375 (2320–13,570)	5510 (3180–19,720)	0.606
Hemoglobin (g/dL)	12.9 (8.6–16.9)	12.1 (7.3–15.0)	0.086
Platelet count (×10^4^/μL)	20.75 (5.2–48.6)	20.9 (6.4–51.1)	0.982
Eosinophil count (/mm^3^)	130 (0–630)	110 (10–880)	0.787
Total bilirubin (mg/dL)	0.7 (0.3–6.8)	0.7 (0.3–1.6)	0.606
Aspartate aminotransferase (IU/L)	24.5 (14–164)	28 (17–73)	0.055
Alanine aminotransferase (IU/L)	18.5 (10–122)	23.0 (7–107)	0.249
Alkaline phosphatase (U/L)	284 (118–2460)	276 (153–833)	0.726
γ-glutamyl transpeptidase (IU/L)	35.5 (10–738)	38.0 (12–505)	0.405
Leucine aminopeptidase (U/L)	60.5 (30–252)	62 (36–254)	0.285
Albumin (g/dL)	4.1 (1.9–5.0)	4.1 (2.4–4.7)	0.387
Autotaxin (mg/L)	1.075 (0.577–3.117)	1.253 (0.719–4.307)	0.309
Estimated glomerular filtration rate (mL/min/1.73 m^2^)	104.6 (22.1–177.5)	70.0 (32.2–164.9)	0.462
HbA1c (%)	5.7 (3.5–7.0)	5.8 (4.9–7.1)	0.765
FIB4 index	1.67 (0.35–10.82)	1.80 (0.52–8.68)	0.769
History of hepatocellular carcinoma (with/without)	4/90	0/25	0.294
History of hepatic encephalopathy or varices (with/without)	0/94	0/25	

Categorical data are represented as numbers of patients, and continuous data are represented as the median and range

## Discussion

Pruritus is a common symptom seen in patients with chronic liver disease. In the present study, 29.8% of the patients with chronic liver disease were evaluated as having ≥1 point pruritus severity by NRS. These frequencies were significantly higher than those of subjects without chronic liver disease. Previous studies reported that pruritus occurs in 5.1–45.9% of HCV-infected patients, 8.2–40.6% of Hepatitis B virus (HBV)-infected patients, and 51.4–69% of PBC patients [21–25]. Furthermore, another study reported that the prevalence of pruritus in AIH, NAFLD, and alcoholic liver disease was 24.3, 44.7, and 34.2%, respectively [21]. Our study showed that the frequencies of pruritus (≥1 point by NRS) were 25.5, 29.7, 33.4, 51.3, 29.8, and 32.6% in patients with hepatitis B, hepatitis C, AIH, PBC, NAFLD, and alcoholic liver disease, respectively. Although there are a few previous reports on frequency of pruritus in patients with AIH, NAFLD, and alcoholic liver disease, our study showed that patients with AIH, NAFLD, and alcoholic liver disease complained of pruritus at similar frequencies to those with hepatitis B or C.

Several hypotheses have been proposed, but the pathogenesis of pruritus in chronic liver disease is complicated and unclear [26]. Thus, it is important to identify the factors associated with pruritus in patients with chronic liver disease. Akuta et al. reported that being HBsAg-negative and having HCC and low platelet count were independent factors associated with severe pruritus in patients with chronic liver disease [27]; on the other hand, Oeda et al. reported that active HBV infection, PBC, diabetes, and AST ≥60 U/L were associated with severe pruritus [21]. In the present study, lower serum albumin level was an independent factor associated with severe pruritus (NRS ≥5 points), suggesting that decrease in liver function could lead to severe pruritus in patients with chronic liver disease. The differences among the predictive markers identified in each study might be due to differences in patient backgrounds, such as history of anti-viral therapy and the state of HCC, as well as differences among the methods used to assess the severity of pruritus and the types of antipruritic agents used.

The frequency of pruritus was higher in PBC patients compared to patients with other etiologies, as is generally known. PBC is a chronic cholestatic liver disease characterized by portal inflammation and immune-mediated destruction of the intrahepatic bile ducts [28], and pruritus is one of the most common symptoms [2]. Our result was consistent with the clinical characteristics of PBC. In the present study, serum autotaxin levels were correlated with platelet count and liver stiffness in PBC patients. Furthermore, autotaxin was correlated with hepatobiliary enzyme levels. These results indicate that autotaxin reflects not only liver fibrosis but also the severity of cholangitis in PBC patients. Wunsch et al. reported that serum autotaxin level increased in patients with PBC or primary sclerosing cholangitis, particularly in patients with cirrhosis and in patients with longer disease duration [20]. It was reported that autotaxin levels are elevated in cholestasis patients with pruritus [16]. In contrast, this study on Japanese PBC patients showed no association between pruritus and liver fibrosis markers, including serum autotaxin, FIB4 index, and hepatobiliary enzyme values. This discrepancy might be due to differences in the assessment of itch severity. Albumin levels were associated with pruritus in all subjects, but not in PBC patients. In patients with PBC, pruritus does not consistently correlate with biological markers of disease [29]. Based on these results, pruritus in PBC patients may be related to genetic factors besides cholestasis and disease progression. Factors associated with pruritus in PBC patients should be further analyzed.

There are some limitations of this study. First, we only investigated hepatic functional reserve and hepatic fibrosis markers such as serum autotaxin levels, FIB4 index, and liver stiffness to isolate factors associated with severe pruritus in PBC patients but not in patients with other etiologies of chronic liver disease. A previous study showed that progression of liver fibrosis was a predictor of pruritus in chronic hepatitis C patients who had achieved viral eradication following DAA therapy [23]. Thus, it is necessary to evaluate the association of liver fibrosis including serum autotaxin levels and pruritus according to the etiology of liver disease. Secondly, we did not perform repeated evaluations of pruritus in patients. Pruritus could vary greatly depending on various factors, including emotional and psychological state and seasonal bias. Follow-up evaluations are required to assess pruritus more accurately in each patient.

## Conclusions

The frequency of pruritus was higher in patients with chronic liver disease than in healthy subjects and patients without chronic liver disease, and decrease in liver function was correlated with severe pruritus based on a large number of patients. Serum autotaxin is useful for assessing liver fibrosis and severity of cholangitis; however, it is not a predictive marker for severe pruritus in patients with PBC.

## Data Availability

All data generated or analysed during this study are included in this published article.

## Abbreviations

AIH: Autoimmune hepatitis
HBV: Hepatitis B virus
HCV: Hepatitis C virus
NAFLD: Non-alcoholic fatty liver disease
NASH: Non-alcoholic steatohepatitis
NRS: Numerical rating scale
PBC: Primary biliary cholangitis
